# Chronic VEGF Blockade Worsens Glomerular Injury in the Remnant Kidney Model

**DOI:** 10.1371/journal.pone.0039580

**Published:** 2012-06-22

**Authors:** Flavia G. Machado, Patrícia Semedo Kuriki, Clarice K. Fujihara, Camilla Fanelli, Simone C. A. Arias, Denise M. A. C. Malheiros, Niels O. S. Camara, Roberto Zatz

**Affiliations:** 1 Laboratory of Renal Pathophysiology (LIM-16), Renal Division, Department of Clinical Medicine, Faculty of Medicine, University of São Paulo, São Paulo, Brazil; 2 Laboratory of Immunology, Nephrology Division, Faculty of Medicine, Federal University of São Paulo, São Paulo, Brazil; The University of Manchester, United Kingdom

## Abstract

VEGF inhibition can promote renal vascular and parenchymal injury, causing proteinuria, hypertension and thrombotic microangiopathy. The mechanisms underlying these side effects are unclear. We investigated the renal effects of the administration, during 45 days, of sunitinib (Su), a VEGF receptor inhibitor, to rats with 5/6 renal ablation (Nx). Adult male Munich-Wistar rats were distributed among groups S+V, sham-operated rats receiving vehicle only; S+Su, S rats given Su, 4 mg/kg/day; Nx+V, Nx rats receiving V; and Nx+Su, Nx rats receiving Su. Su caused no change in Group S. Seven and 45 days after renal ablation, renal cortical interstitium was expanded, in association with rarefaction of peritubular capillaries. Su did not worsen hypertension, proteinuria or interstitial expansion, nor did it affect capillary rarefaction, suggesting little angiogenic activity in this model. Nx animals exhibited glomerulosclerosis (GS), which was aggravated by Su. This effect could not be explained by podocyte damage, nor could it be ascribed to tuft hypertrophy or hyperplasia. GS may have derived from organization of capillary microthrombi, frequently observed in Group Nx+Su. Treatment with Su did not reduce the fractional glomerular endothelial area, suggesting functional rather than structural cell injury. Chronic VEGF inhibition has little effect on normal rats, but can affect glomerular endothelium when renal damage is already present.

## Introduction

VEGF is one of the most important proangiogenic factors, exerting a potent mitogenic activity on endothelial cells [1]–[4]. In the kidneys, VEGF is produced predominantly in podocytes, in the distal tubule and collecting duct, and, to a lesser extent, in the proximal tubule [5]. In addition to its paracrine effects on the glomerular endothelium, VEGF produced by podocytes may exert an autocrine action, substantially influencing the survival and integrity of the podocyte itself [6].

VEGF inhibition with drugs such as bevacizumab and VEGF-Trap has been widely used to limit the growth of solid tumors by restricting their blood supply [7]. In addition, VEGF action can be inhibited by inactivating the tyrosine kinase domain of its receptors with broad-spectrum drugs such as sunitinib, sorafenib and vatalanib [7]–[9].

Therapies that target VEGF bring a number of adverse effects, of which proteinuria, hypertension and thrombotic microangiopathy are the most commonly observed [10]. However, the mechanisms involved in the pathogenesis of these toxic effects are unclear. Reduction of angiogenic activity in the renal parenchyma, with development or aggravation of tissue hypoxia, may promote interstitial inflammation [11]–[13], thus favoring the development of hypertension [14]. In addition, inhibition of VEGF paracrine action on the glomerular endothelium may lead to alterations of the endothelial surface, favoring the development of thrombotic microangiopathy. Finally, there remains the possibility that the deleterious effect of VEGF inhibitors may be due to a toxic effect on podocytes, as a result of the abrogation of the presumed autocrine action of VEGF [6], [15]. The incidence of such adverse events is extremely variable, depending on the drug used, its dosage, the underlying disease and duration of treatment, among several factors [7], [10], [16]. A possible risk factor facilitating the development of these side effects is the presence of underlying renal dysfunction. However, this possibility has not been examined.

In the present study, we investigated the renal structural and functional effects of the administration of sunitinib up to 45 days. Although the effects of this treatment were minimal in normal rats, the drug promoted significant worsening of the glomerular changes associated with 5/6 renal ablation, a well-known model of chronic renal disease.

## Results

Mortality was very low in this study, with only one death in the treated Nx group, and none in the remaining experimental groups. The results for body weight (BW), TCP, U_alb_V, S_Cr_ and arterial hematocrit (Ht) 7 and 45 days after renal ablation are presented in Table 1. All groups gained weight throughout the study. However, body growth was slower in both groups of nephrectomized animals. The S+Su animals exhibited a slight but significant limitation of body growth at the end of the study. The treatment with Su did not significantly affect the growth of nephrectomized animals.

**Table 1:** Renal and systemic parameters 7 and 45 days after renal ablation.

		BW	TCP	U_alb_V	Ht	S_Cr_
**Day 7**	**S+V**	239±2	136±2	2.4±0.5	47±1	0.50±0.02
	**S+Su**	238±3	139±1	2.1±0.4	44±1	0.53±0.02
	**Nx+V**	208±2^a^	167±4^a^	55.5±13.6^a^	49±1	1.16±0.04^a^
	**Nx+Su**	206±4^a^	165±3^a^	53.6±10.8^a^	46±1	1.26±0.07^a^
**Day 45**	**S+V**	322±4^c^	139±2	4.9±1.4	48±1	0.59±0.02
	**S+Su**	301±5^bc^	140±4	6.8±3.2	48±1^c^	0.54±0.02
	**Nx+V**	256±7^ac^	211±4^ac^	134.1±15.1^ac^	44±1^c^	1.19±0.07^a^
	**Nx+Su**	250±7^ac^	213±3^ac^	149.1±14.3^ac^	39±2^abc^	1.46±0.14^ab^

Mean ±1 SE; BW: body weight, grams; TCP: tail-cuff pressure, mmHg; U_alb_V: daily urinary albumin excretion rate, mg/24 h; Ht, arterial hematocrit, %; S_Cr_: serum creatinine concentration, mg/dL. ^a^p<0.05 vs. respective S; ^b^p<0.05 vs. respective untreated; ^c^p<0.05 vs. respective value on Day 7.

TCP was stable in sham-operated rats throughout the study (Table 1). By contrast, Nx animals exhibited a progressive elevation of TCP that was already apparent 7 days after renal mass removal. Treatment with Su did not aggravate hypertension in Nx animals. U_alb_V remained at low levels in sham-operated rats during the study, and was unaffected by Su treatment (Table 1). Nx rats exhibited a marked elevation of U_alb_V with time. Su treatment of Nx rats promoted no statistical change in U_alb_V.

S_Cr_ remained stable in S rats, and was not affected by Su treatment (Table 1). In Nx rats, S_Cr_ was expectedly elevated compared to Group S, but remained stable during the study. In Group Nx+Su, S_Cr_ elevation was similar to that in untreated Nx rats at 7 days, but increased further at 45 days, after renal ablation.

Su treatment did not affect Ht in S rats (Table 1). Ht was expectedly reduced in untreated Nx at 45 days. Su treatment promoted an additional decrease in Ht at this time point.

**Figure 1:**
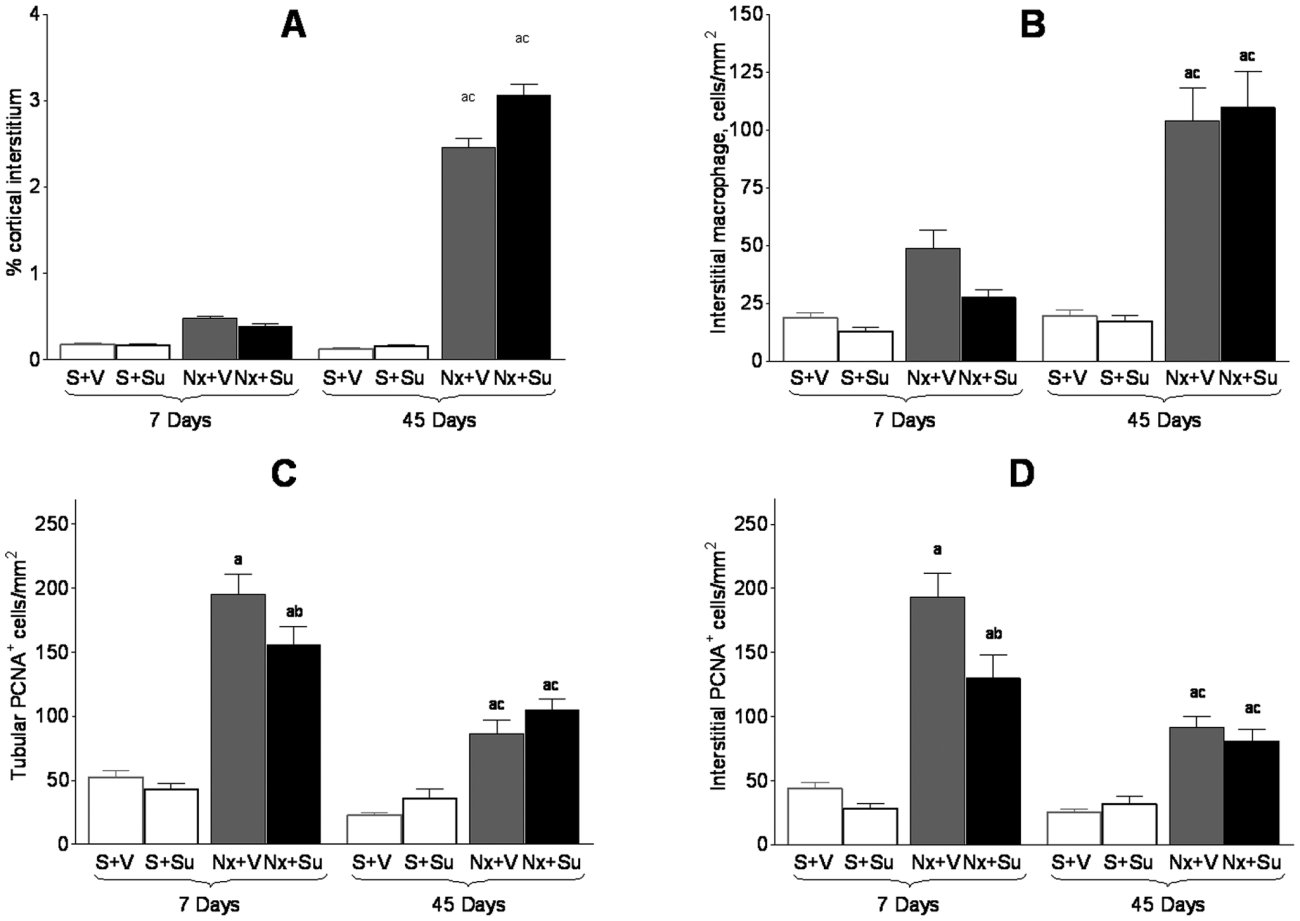
Evaluation of tubulointerstitial changes. A) Fractional cortical interstitial area; B) Cortical interstitial infiltration by macrophages; C) Tubular cells in proliferation (PCNA^+^); D) Interstitial cells in proliferation (PCNA^+^);^ a^p<0.05 vs. respective S; ^b^p<0.05 vs. respective untreated; ^c^p<0.05 vs. respective value on Day 7.

Seven days after renal ablation, Nx rats showed only a small numerical increase of the fractional cortical interstitial area (%INT) compared to S (Figure 1A). On Day 45, Nx animals showed a marked increase in %INT compared to S. No change of %INT was induced by Su treatment in either S or Nx at any time.

The intensity of macrophage (MØ) infiltration in the renal cortical interstitium is shown in Figure1B. There was no significant difference between groups S+V and S+Su. Nx rats exhibited only a numerical increase of cortical interstitial MØ density 7 days after renal ablation, compared with their respective controls. At 45 days, Nx rats showed an intense and significant interstitial MØ infiltration compared with S, as well as with the value observed at 7 days. Su treatment did not alter the density of cortical interstitial MØ infiltration in Nx animals at any time.

The proliferation of tubular and interstitial cells (Figures 1C and 1D) was similar between S+V and S+Su, at both 7 and 45 days of treatment. In Nx rats there was a marked increase in the number of tubular and interstitial PCNA-positive cells at 7 days after renal ablation. At this time, Su treatment promoted a moderate but significant reduction of tubular and interstitial proliferation in Nx rats. At 45 days, the proliferative activity abated in Nx rats, but remained elevated compared with S. Treatment with Su did not change this parameter at this point in time.

**Figure 2:**
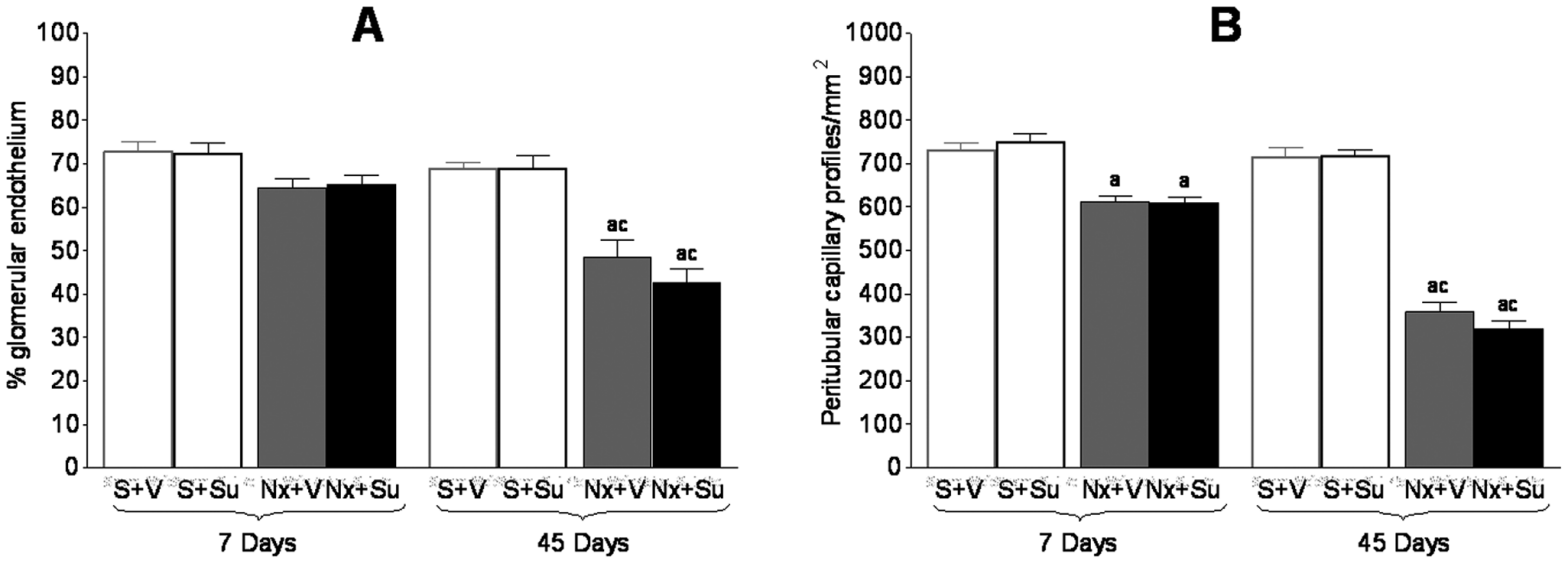
Vascular endothelial evaluation. A) % glomerular area occupied by endothelium; B) Number of peritubular capillary profiles. ^a^p<0.05 vs. respective S; ^b^p<0.05 vs. respective untreated; ^c^p<0.05 vs. respective value on Day 7.

The area occupied by glomerular endothelium, as assessed by detection of the JG-12 antigen, is shown in Figure 2A. S+Su rats showed no difference in the glomerular endothelial area compared to the respective untreated group. We observed only a numerical difference between Nx and S rats 7 days after renal ablation. However, this difference became significant at 45 days, indicating a progressive loss of glomerular endothelium. Treatment with Su did not alter significantly the glomerular endothelial area in Nx animals, at either 7 or 45 days.

The density of peritubular capillaries in the renal cortex, estimated by quantification of the endothelium-specific JG-12 antigen, decreased progressively in Nx rats (Figure 2B). Treatment with Su did not result in a significant change of peritubular vascularization in either S or Nx rats.

**Figure 3:**
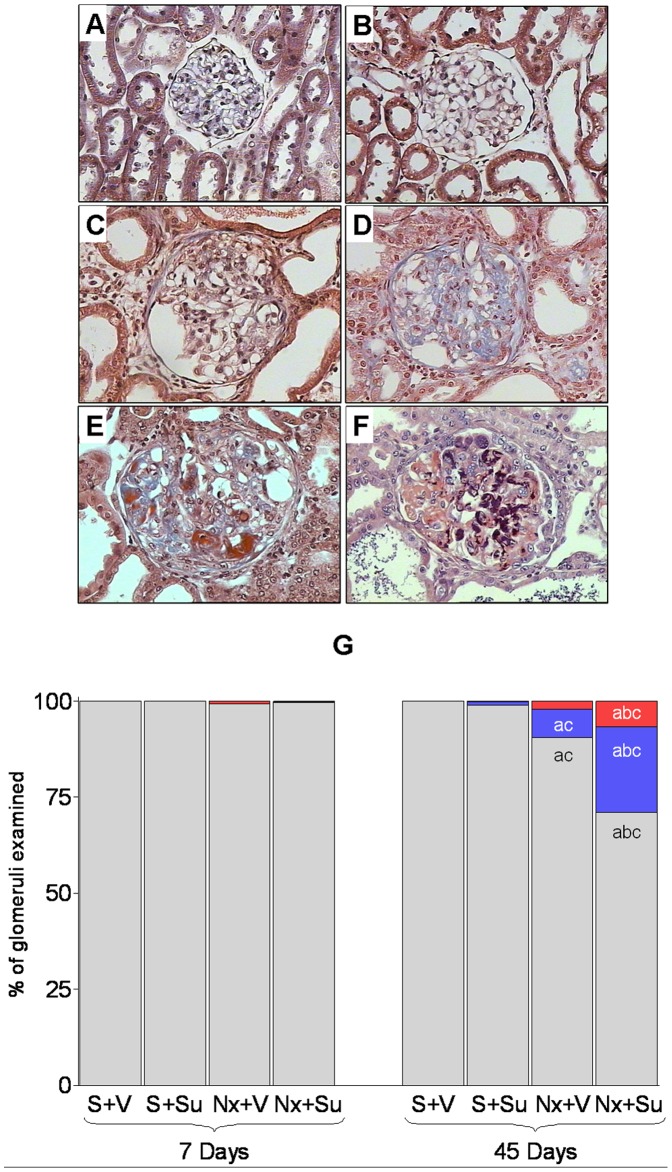
Histologic and histomorphometric analysis of glomerular injury (Masson Trichrome). A) and B) normal glomeruli found in Group S+V and S+Su, respectively; C) and D) representative glomerular sclerotic lesions found in Group Nx+V and Nx+ Su. E) glomerular intracapillary microthrombi observed in Group Nx+Su, some of which appear partially organized. F) PTAH staining showing the presence of fibrin (in dark purple) in glomerular capillary lumina of Nx+Su rats. G) distribution of glomerular lesions among “microthrombi” (red) and “sclerosis” (blue). Normal glomeruli are represented in gray. ^a^p<0.05 vs. respective S; ^b^p<0.05 vs. respective untreated; ^c^p<0.05 vs. respective value on Day 7.

On Day 7 after renal ablation, no glomerular abnormalities were seen in either S+V or S+Su rats, 100% of glomeruli in these groups being classified as normal (Figure 3A and 3B). This pattern was maintained at Day 45, despite the presence of rare glomerular microaneurysms in Group S+Su. At 7 days of treatment, occasional sclerotic lesions and intracapillary microthrombi, were seen in a small amount of glomeruli from Groups Nx+V and Nx+Su (p>0.05 compared to respective controls). The frequency of these abnormalities was significantly increased on Day 45 (Figures 3C and 3D), especially in Group Nx+Su, in which 7% of glomeruli showed intracapillary microthrombi (Figure 3E and 3F), most of which appeared partially organized (Figure 3E), whereas 22% exhibited segmental or diffuse sclerotic lesions (Figure 3G, p<0.05 NX+Su vs. Nx+V). Microthrombi were also occasionally observed in the lumen of renal arterioles in Group Nx+Su (not shown).

**Figure 4:**
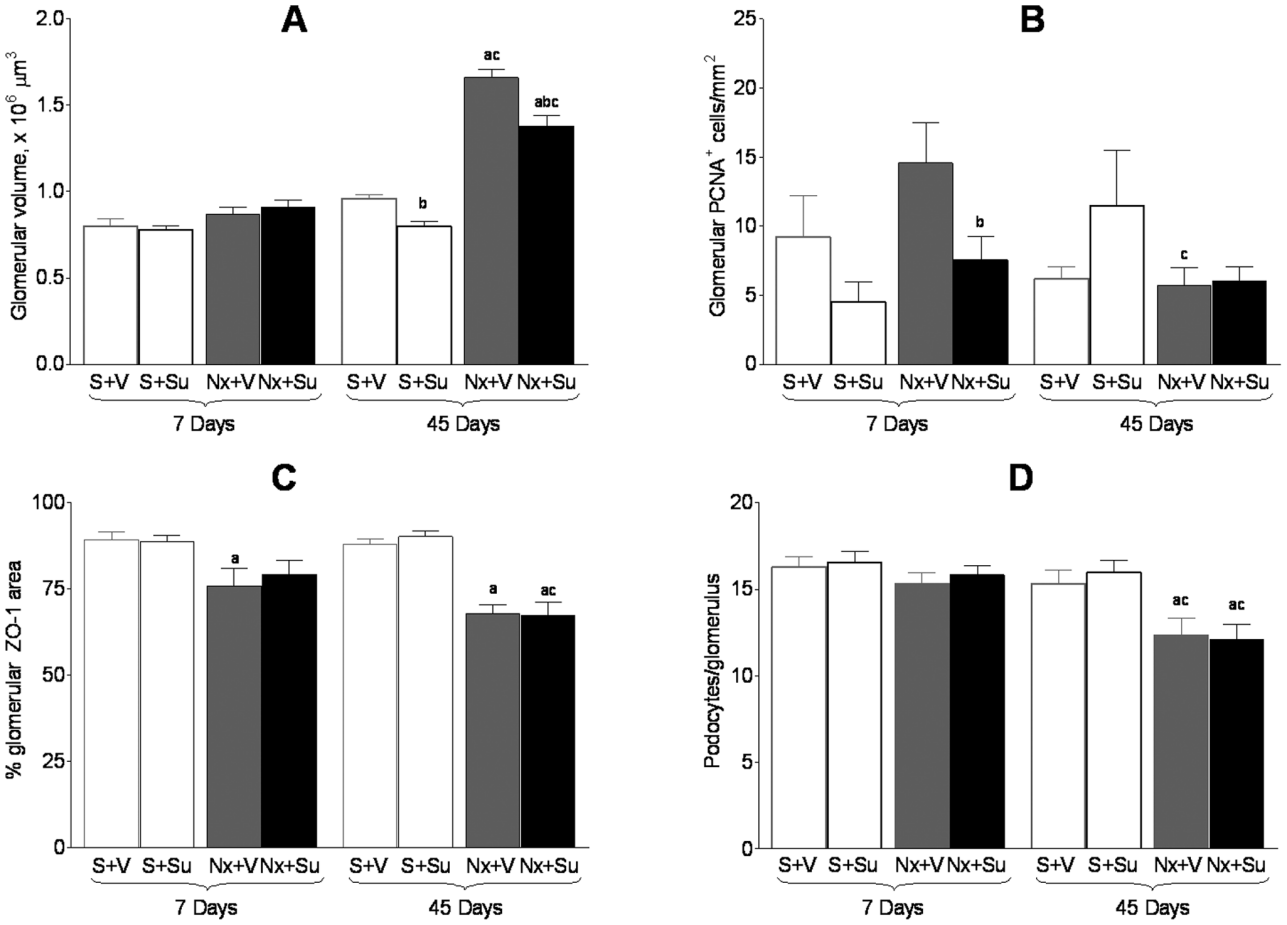
A) Glomerular volume; B) glomerular cell proliferation assessed by the number of PCNA^+^ cells; C) Percentage of glomerular area staining positively for ZO-1; D) Number of podocytes per glomerulus. ^a^p<0.05 vs. respective S; ^b^p<0.05 vs. respective untreated; ^c^p<0.05 vs. respective value on Day 7.

No significant difference in glomerular volume was seen between S and Nx groups 7 days after renal ablation (Figure 4A). At 45 days, Nx+V exhibited a marked increase in glomerular volume, in agreement with previous studies. Su treatment attenuated glomerular hypertrophy, although tuft volume in Group Nx+Su was still significantly larger than in Group S+V.

The amount of proliferating cells, detected by staining for PCNA, was much lower in the glomerular tuft than in tubules or in the cortical interstitium. On Day 7 after renal ablation, no statistically significant difference in the frequency of glomerular proliferating cells was seen between S and Nx rats (Figure 4B). Treatment with Su had no effect in S, but lowered the amount of PCNA-positive cells in Nx. At 45 days, the extent of cell proliferation at the glomeruli remained low compared with either tubules or cortical interstitium. No effect of Su treatment was detected at this time.

No difference in the percentage of glomerular area staining positively for ZO-1 was seen between Groups S+V and S+Su, at either 7 or 45 days after renal ablation (Figure 4C). However, Nx rats showed a persistent reduction in the glomerular staining for ZO-1, indicating profound structural changes of podocytes. Treatment with Su did not result in additional changes of this parameter, no significant difference being observed between Groups Nx+V and Nx+Su at either 7 or 45 days of treatment.

The number of podocytes per glomerulus, assessed by the detection of the WT-1 antigen by immunohistochemistry was significantly reduced in Group Nx 45 days after renal ablation (Figure 4D). The number of podocytes per glomerulus was not affected by treatment with Su in either S rats or Nx rats.

**Figure 5:**
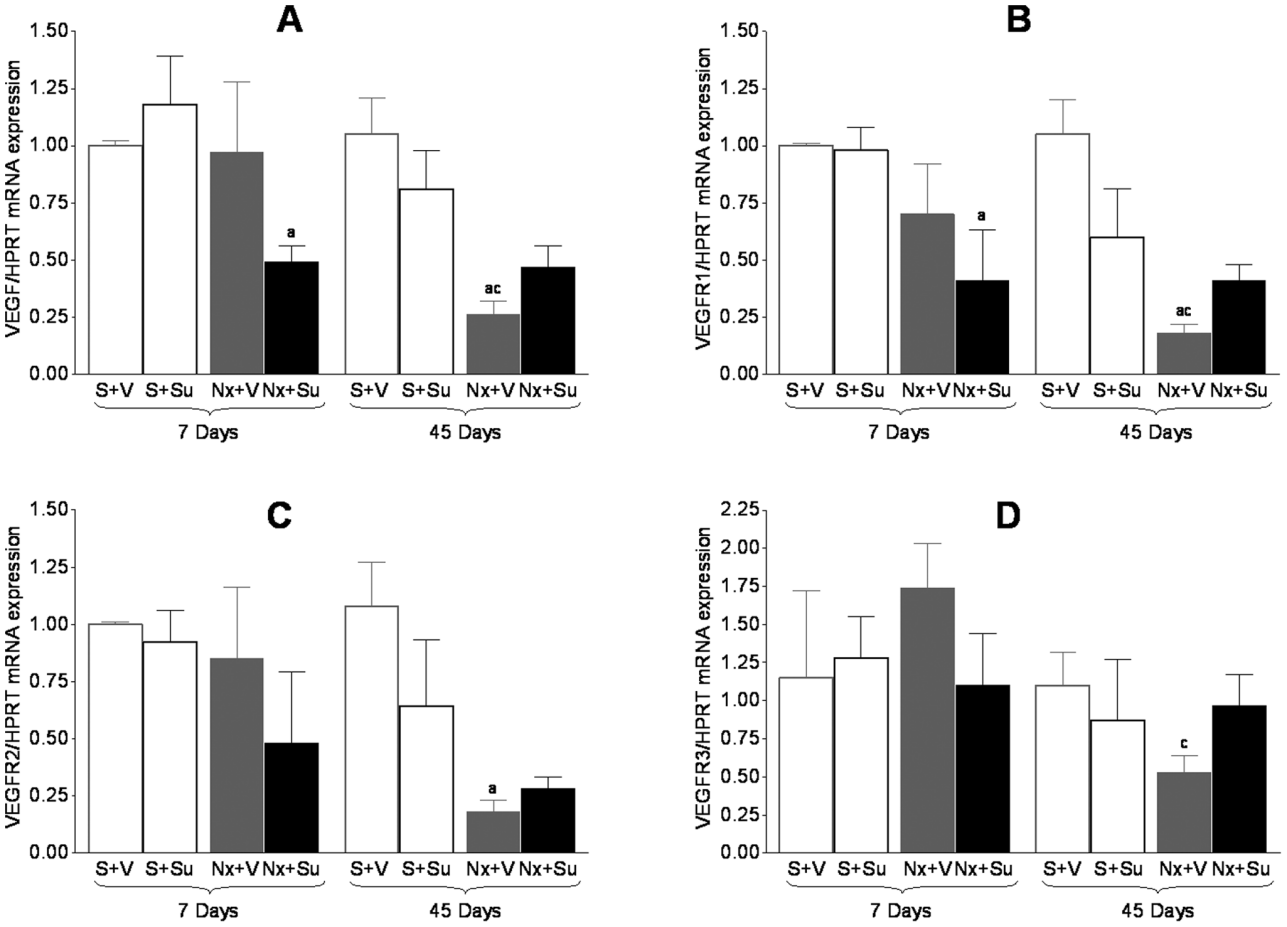
RT-PCR analysis of the expression of VEGF (A) and of its receptors, VEGFR1 (B), VEGFR2 (C), and VEGFR3 (D). ^a^p<0.05 vs. respective S; ^b^p<0.05 vs. respective untreated; ^c^p<0.05 vs. respective value on Day 7.

The gene expression of VEGF and of its receptors (VEGFR1, VEGFR2 and VEGFR3) was assessed by RT-PCR in real time at 7 and 45 days. No difference between S and Nx+V animals was seen 7 days after renal ablation (Figure 5). However, Nx rats treated with Su showed VEGF downregulation in renal tissue at this time compared to the untreated Nx group. After 45 days, both Nx+V and Nx+Su rats exhibited a significant reduction in the renal expression of VEGF and of its receptors, no significant difference being observed between these two groups. No effect of Su treatment was seen in S rats.

## Discussion

VEGF is a key element in physiologic and pathologic processes such as wound healing and neoplastic growth. VEGF contributes decisively to the homeostasis and survival of endothelial cells, inhibiting apoptosis even under stressful conditions [17], [18]. Adequate VEGF levels are also essential for placental homeostasis and, accordingly, experimental preeclampsia is associated with low VEGF activity [19]. VEGF is produced by renal tubular epithelial cells [20], [21], and may thus contribute to ensure adequate supply of oxygen for solute transport. In glomeruli, VEGF is mostly generated by the podocytes, exerting a crucial paracrine effect on endothelial cells [15], [22]. Accordingly, administration of VEGF inhibitors as part of antineoplastic chemotherapy has been associated with the development of reversible hypertension and proteinuria [7], [10], [16] and/or glomerular microthrombosis [23], [24].

Unlike the reports of toxicity after human use of sunitinib, we found no hypertension, proteinuria or histologically detectable glomerular damage in sham-operated rats treated with sunitinib (Group S+Su), which was administered at a similar dose per body surface as the maximum dose used in humans. These findings are also apparently in disagreement with those obtained by Kappers, et al [25], who reported blood pressure elevation in rats treated with sunitinib. However, it must be noted that these investigators utilized much higher doses of the drug per m^2^ of body surface than employed in the present study and in human subjects.

Despite its lack of effect on blood pressure, sunitinib was not innocuous when administered to S rats. First, body growth rate was significantly lower in Group S+Su than in Group S, indicating that the drug did exert a biological effect. Second, and in accordance with previous clinical observations [26], [27], the administration of sunitinib to Nx animals resulted in a decrease in hematocrit, not unexpected considering the known influence of VEGF on hematopoiesis [28], [29] and the low renal expression of VEGF observed in this group. The lack of effect of Su on blood pressure or proteinuria was paralleled by an equally undetectable effect on the density of endothelial cells, either in glomeruli or peritubular capillaries, at odds with the well-known anti-angiogenic action of the drug. This finding is in keeping with the notion that the turnover of endothelial cells is rather low in normal renal tissue [30], [31].

Five-sixths renal ablation represents a context of intense structural and functional alteration at both the glomeruli and the renal interstitium. As shown in previous studies [32], [33] and corroborated by the present observations, Nx rats exhibit a peak of cell proliferation 7 days after renal ablation, which is contributed, at least in part, by tubular epithelial cells and myofibroblasts [34]. Although some previous studies showed that endothelial cells can also contribute to this initial proliferative surge [30], [31], we observed, as early as 7 days after renal ablation, a modest but significant reduction in the density of cells staining positively for the JG-12 antigen, specific for vascular endothelial cells. This observation is consistent with our finding that the renal expression of VEGF, as well as of its receptors, was diminished in Nx rats at this time, corroborating previous findings [30], [35], [36] and suggesting that the participation of endothelial cells in the intense proliferative activity observed at this early stage is rather limited. Also in accordance with previous studies [30], [31], peritubular capillary density was progressively rarefied in Nx rats, which may have contributed, along with the observed distortion of the interstitial architecture, to the development of tissue hypoxia and the progression of renal injury [37].

Chronic treatment with Su promoted a modest reduction in interstitial cell proliferation 7 days after ablation. However, it is unlikely that this finding reflects a specific antiproliferative effect of Su treatment on endothelial cells, since the density of peritubular capillaries in Group Nx+Su never differed significantly from that observed in untreated Nx rats, indicating that the impairment of the renal microcirculation by renal ablation was not aggravated by the drug. Accordingly, Su treatment did not worsen renal interstitial expansion/inflammation in Nx rats. In addition, Su treatment affected neither the renal expression of VEGF nor that of its receptors. Together, these observations indicate that chronic Su treatment of Nx rats had little impact on the tubulointerstitial perfusion or on the extent of structural damage to the renal interstitium. However, we cannot exclude the possibility that a significant effect of Su treatment on these parameters would be seen if a longer period of observation were feasible.

In contrast with its little effect on the renal interstitium, Su treatment exerted a considerable impact on the glomeruli of Nx rats, in which a marked increase in the frequency of sclerotic lesions was observed 45 days after renal ablation. The mechanisms involved in the exacerbation of glomerulosclerosis by Su are not immediately apparent. Mechanical stress, caused by glomerular hypertension and/or glomerular hypertrophy, plays an important role in the pathogenesis of progressive glomerulosclerosis [38], [39]. Glomerular tuft volume was indeed increased in Nx rats. Su treatment of these animals did not increase glomerular volume at 7 days, and even attenuated tuft hypertrophy at the end of the experimental protocol. These findings are in keeping with those obtained in Nx [40] and uninephrectomized [41] rats by neutralizing VEGF activity. The possibility that the deleterious effect of Su was due to exacerbation of intraglomerular hypertension, a well-known factor of glomerular injury, cannot be excluded, since glomerular hydraulic pressure was not measured in this study. However, this explanation seems unlikely, since glomerular hypertension and glomerular hypertrophy are invariably associated in the Nx model [33], [38], [39], [42]. A possible exacerbation of glomerular hyperplasia is also unlikely, given that Su treatment did not alter the rate of cell proliferation in the glomerular tuft, and even reduced it on Day 7. The latter effect may have resulted from a possible action of Su on mesangial PDGF receptors [9], which are known to stimulate glomerular cell proliferation [32] and to promote progressive glomerular injury [43]. Since sunitinib treatment accelerated glomerulosclerosis, rather than ameliorating glomerular injury, any renoprotection that might have been afforded by PDGF inhibition must have been surpaseded by the effect of the drug on VEGF receptors.

Podocyte loss or injury is one of the possible mechanisms underlying the development of glomerulosclerosis in renal mass removal models [44], [45]. In the present study, the number of podocytes was clearly diminished in Nx rats, as evidenced by the progressive decrease, compared with Sham rats, of the glomerular content of the WT-1 antigen, while the deficiency of ZO-1 indicates that the connection between podocytes and the integrity of the slit membrane, hence the structure of the glomerular visceral epithelial layer, were compromised in these animals. Podocyte damage in the Nx model may be due to mechanical stress from glomerular hypertension and/or hypertrophy. Additional injury may result from the declining renal levels of VEGF in these rats, since VEGF, which is mainly produced by podocytes [20], [46], may exert a trophic autocrine effect on these cells [6], [47]. However, administration of Su to Nx rats, caused no additional damage to these cells, as indicated by the little effect on albuminuria, and by the lack of further changes in the renal content of WT-1 or ZO-1 in Group Nx+Su. These findings lend no support to the concept that VEGF exerts a vital autocrine effect on podocytes, and indicate that the aggravation of GS by Su treatment cannot be explained by an exacerbation of podocyte injury. Accordingly, these results are consistent with recent observations, in mice with podocyte-specific deletion of the VEGFR-2 receptor, that a VEGF-VEGFR2 interaction is absent in podocytes [22].

Abundant evidence now indicates that VEGF, through its binding to VEGFR2, exerts a paracrine effect on the glomerular endothelium, which is crucial to the proliferation, differentiation and survival of these cells [15], [22], [48]. Abrogation of this trophic effect may help to explain the glomerular changes associated with the use of anti-angiogenic therapies based on VEGF inhibition [10], [23], [49] and reproduced in studies in which targeted silencing of the VEGF gene was specifically performed in podocytes [23]. These adverse effects consist mostly of proteinuria and hypertension [7], [10]. However, the formation of microthrombi in glomerular capillaries is also frequently observed in patients treated with VEGF inhibitors and in experimental studies of specific deletion of the VEGF gene, suggesting damage to the vascular endothelium [23], [24], [49]. The presence of focal segmental glomerulosclerosis has occasionally been demonstrated in these patients [49], [50]. Nevertheless, we were unable to detect the presence of microthrombi in sunitinib-treated sham-operated rats, although a few glomerular microaneurysms were observed in these animals.

Nx rats exhibited a decline in the area occupied by glomerular endothelium. This finding, which corroborates previous observations [30], may result from mechanical stress caused by glomerular hypertension and hypertrophy, and/or from the decreased renal expression of VEGF, observed in this study as well as in other studies of the Nx model [30], [35]. Damage and/or loss of endothelial cells likely explain the presence of microthrombi in Nx rats, which affected about 2% of the examined glomeruli. Blockade of VEGF receptors with sunitinib exacerbated the formation of glomerular microthrombi, the frequency of which nearly tripled. Since sunitinib treatment promoted no additional loss of glomerular endothelial cells in Nx rats, formation of microthrombi may reflect functional changes of the endothelial cells, as suggested by previous studies [23], [24], [49], [50], resulting in alteration of their surface and facilitating platelet adhesion. Organization of microthrombi, suggested by the present histologic observations, may explain the aggravation of glomerulosclerosis in sunitinib-treated Nx rats in the absence of additional podocyte damage or exacerbation of proteinuria.

An unexpected finding of this study was that the expression of both VEGF and of its main receptors was downregulated in the renal tissue of Nx rats. The reason for this finding is unknown. VEGFR2 has been demonstrated to be absent in podocytes [22] and, accordingly, sunitinib had little effect on these cells. These observations indicate that any effect of the drug on VEGF production must have taken place outside the glomeruli, that is, in tubules and/or pericytes. However, any mechanistic interpretation of these findings is necessarily speculative before further data becomes available.

In summary, the results of the present study suggest that VEGF inhibition by sunitinib does not cause gross alterations in the glomerular endothelium or in the renal structure of normal rats. However, when nephron number is reduced, the action of the drug may add to that of other pathogenic factors such as glomerular hemodynamic stress, promoting glomerular changes that culminate in the formation of intracapillary microthrombi. Functional changes of the glomerular endothelium, with subsequent organization of microthrombi, may explain the worsening of glomerulosclerosis observed in animals treated with the drug, although the present data do not warrant a firm conclusion in this regard. Renal ablation resulted in progressive capillary rarefaction at the cortical interstitium. Administration of Su did not aggravate tubulointerstitial fibrosis or the loss of peritubular capillaries in Nx rats, indicating that the degree of compensatory angiogenesis must be low in this model.

## Materials and Methods

### Experimental Groups

Adult male Munich-Wistar rats (n = 151), weighing 220–260 g, obtained from a local facility at the Faculty of Medicine, University of São Paulo, were used in this study. Five-sixths renal ablation (Nx) was performed in a single step procedure after ventral laparotomy under anesthesia with ketamine 50 mg/kg and xylazine 10 mg/kg im. The right kidney was removed and two branches of the left renal artery were ligated, resulting in the infarction of two-thirds of the left kidney. The whole operation lasted about 30 minutes. Sham-operated rats underwent anesthesia and manipulation of the renal pedicles, without any removal of renal mass. Rats were then returned to their cages and were fully recovered and active three hours after surgery. All animals were given free access to tap water, fed regular rodent chow containing 0.5 Na and 22% protein (Nuvital Labs, Curitiba, Brazil), and kept at 23±1°C and 60±5% relative air humidity, under an artificial 12–12 hour light/dark cycle. All experimental procedures were specifically approved by the local Research Ethics Committee (Comissão de Ética para Análise de Projetos de Pesquisa do Hospital das Clínicas da Faculdade de Medicina da Universidade de São Paulo, CAPPesq under process n° 0166/07), and developed in strict conformity with our institutional guidelines and with international standards for manipulation and care of laboratory animals.

### Experimental Groups

Sunitinib treatment was started on the day after renal ablation. The drug was dissolved in carboxymethylcellulose 0.5% at a concentration of 2 mg/mL and 2 mL/kg of this solution was administered by gavage once daily, so as to achieve a dosage of 4 mg/kg/day. Sham and Nx rats were distributed among four groups: S+V, sham-operated rats receiving vehicle (carboxymethylcellulose) only (N = 31); S+Su, sham-operated rats receiving sunitinib as described earlier (N = 32); Nx+V, Nx rats treated with vehicle only (N = 43); and Nx+Su, Nx rats treated with sunitinib as detailed above (N = 45).

### Renal Morphology Evaluation

At either 7 or 45 days of treatment, 24-h urine was obtained for assessment of albumin (U_alb_V) by radial immunodiffusion. The tail-cuff pressure (TCP) was measured using an optoelectronic automated device (Visitech Systems, Apex, NC), under light restraining and after light warming. To avoid any interference of stress, all rats were preconditioned to the procedure, and were invariably calm at the time of TCP determination. In addition, blood pressure evaluation was always performed after stabilization, that is, TCP was taken as the average of at least three consecutive measurements that varied by no more than 2 mmHg. Rats were then anesthetized with ketamine, 50 mg/kg and xylazine 10 mg/kg im, and a 1-mL blood sample was collected from the abdominal aorta for measurement of hematocrit and serum creatinine concentration (S_Cr_). The kidneys were then retrogradely perfusion-fixed through the abdominal aorta with Dubosq-Brazil solution after a brief washout with saline to remove blood from the renal vessels. After weighing, two midcoronal renal slices were postfixed in buffered 4% formaldehyde and embedded in paraffin using conventional sequential techniques. Histomorphometric and immunohistochemical analyses of the renal tissue were performed in 4-µm-thick sections.

### Histomorphometric Analysis

All histomorphometric evaluations were performed in Masson trichrome-stained sections by a single observer who was blinded to the experimental groups. The frequency of glomerulosclerotic lesions and microthrombi was determined in at least 60 glomeruli per rat (>100 in over 90% of all animals). The fraction of the renal cortical area occupied by interstitial tissue (%INT), used as a measure of the degree of interstitial expansion, was estimated by a point counting technique [51]. The presence of fibrin in the lumina of glomerular capillaries was detected by staining with phosphotungstic acid hematoxylin (PTAH).

Mean glomerular random cross sectional area (A_G_) was determined by averaging individual values for 25 consecutive glomerular tuft profiles using an image processing software (Image Pro Plus®, version 7.01). The average glomerular tuft volume (V_G_) for each rat was then calculated as V_G_ = 1.25 (A_G_)^3/2^ [52].

### Immunohistochemical Analysis

Immunohistochemistry was performed on 4-µm-thick sections, mounted on glass slides precoated with 2% silane. Sections were deparaffinized and rehydrated by conventional techniques, then heated in citrate buffer for antigen retrieval and incubated overnight with the primary antibody at 4°C. For the negative control experiments, incubation with the primary antibody was omitted. The following primary antibodies were used: monoclonal mouse anti-rat ED-1 antibody (for macrophage detection; Serotec, Oxford, United Kingdom); monoclonal mouse anti-rat proliferating cell nuclear antigen (PCNA) (Dako, Glostrup, Denmark); monoclonal mouse anti-rat endothelial aminopeptidase P (JG-12) (Bender MedSystems, California, USA); polyclonal rabbit anti-human zonula occludens-1 (ZO-1) (Zymed, San Francisco, USA); and monoclonal mouse anti-human Wilms’ tumor 1 (WT-1) (Dako, Glostrup, Denmark).

For ED-1 detection, sections were preincubated with 5% normal rabbit serum to prevent nonspecific binding, then incubated overnight at 4°C with the primary antibody, diluted in bovine serum albumin (BSA) at 0.5%. After rinsing with Tris-buffered saline (TBS), sections were incubated with a 2% solution of rabbit anti-mouse immunoglobulin (Dako, Glostrup, Denmark) in BSA, then with an alkaline phosphatase anti-alkaline phosphatase (APAAP) complex (Dako, Glostrup, Denmark). Sections were then developed with a fast-red dye solution (Sigma-Aldrich, Saint Louis, MO). For detection of all other antigens, sections were pretreated with 30% hydrogen peroxide in methanol and preincubated with normal horse serum diluted in 2% non fat milk. The primary antibodies were also diluted in 2% non fat milk. The Envision System (Dako, Glostrup, Denmark) was used for PCNA detection, whereas the NovoLink Polymer Detection System (Novocastra, Benton Lane, United Kingdom) was utilized for the remaining antigens. Sections were developed with DAB substrate (Dako, Glostrup, Denmark).

All slices were counterstained with Mayer’s hemalaum and covered with a glycerin-gelatin mixture.

The renal density of macrophages and proliferating cells was evaluated in a blinded manner at ×200 magnification. For each section, 25 microscopic fields (corresponding to a total area of 1.6 mm^2^) were examined. The percentage of glomerular area staining positively for ZO-1 or JG-12 was evaluated with an image processing software (Image Pro Plus®, version 7.01). The number of podocytes per glomerular tuft was assessed in 25 glomeruli per rat under 400× magnification. Peritubular capillaries were counted in 25 consecutive microscopic fields under a 200× magnification and expressed as the number of capillary profiles per mm^2^ of cortical tissue.

### Gene Expression

Renal tissue was collected at 7 and 45 days, being quickly frozen in liquid nitrogen. Total RNA was isolated using TRIzol Reagent (Invitrogen, Carlsbad, USA), and RNA concentrations were determined by Nanodrop (ND-1000 UV-Vis). First-strand cDNAs were synthesized using the MML-V reverse transcriptase (Invitrogen, Carlsbad, USA). Reverse transcription-polymerase chain reaction (RT-PCR) for VEGF-A (*Rn 00582935_m1*) and for HPRT (*Rn01527838_g1*) was performed using TaqMan probes (Applied Biosystems, Foster City, USA) at ABI PRISM 7300 Sequence Detector, using Sequence Detection Software 1.9 for analysis (Applied Biosystems, Foster City, USA). The following primers were designed based on a known sequence of nucleobases in GenBank, being adapted for the PCR technique by Primer Express software (Applied Biosystems, Foster City, USA): for VEGFR1/Flt1 *sense* 5′-GACAAGGGACTCTACACTTGTCGT-3′, *antisense* 5′-CGATGCTTCACGCTGATAAATCCC-3′; for VEGFR2/Flk1 *sense* 5′-ACTACACGGTCATCCTCACCAATC-3′, *antisense* 5′-AGGAGAGATCAAGGCTTTCTCACC-3′; for VEGFR3/Flt4 *sense* 5′- AAGGAAGCTTCTTCACCCAGCATC-3′, *antisense* 5′- GGCAAATGTCTTACAGGGTGTCCA-3′. For PCR Real Time amplification, Mix TaqMan (Applied Biosystems, Foster City, USA), or SYBR Green PCR Master Mix (Applied Biosystems, Foster City, USA) were used. Cycling conditions for SYBR primers were 10 min at 95°C followed by 45 cycles of 20 sec at 95°C and 20 sec at 60°C. mRNA expression was normalized to HPRT (housekeeping gene) abundance. The Ct (threshold cycle) for the target gene and the Ct for the internal control were determined for each sample and the relative mRNA expression was calculated by the 2^−ΔΔCT^ method. All RT-PCR experimental results are expressed as an n-fold difference relative to the calibrator. All samples were analyzed in triplicate.

### Statistical Analysis

Statistical differences were assessed by two-way ANOVA, with treatment and time as intervening factors and with pairwise posttest comparisons according to the Bonferroni method [53]. Gene expression results were assessed by one-way ANOVA with pairwise posttest comparisons according to the Bonferroni method [53]. Results are expressed as Mean ± SE.
